# High performance visible-SWIR flexible photodetector based on large-area InGaAs/InP PIN structure

**DOI:** 10.1038/s41598-022-11946-7

**Published:** 2022-05-10

**Authors:** Xuanzhang Li, Junyang Zhang, Chen Yue, Xiansheng Tang, Zhendong Gao, Yang Jiang, Chunhua Du, Zhen Deng, Haiqiang Jia, Wenxin Wang, Hong Chen

**Affiliations:** 1 Key Laboratory for Renewable Energy, Beijing Key Laboratory for New Energy Materials and Devices, Beijing National Laboratory for Condensed Matter Physics, Institute of Physics, Chinese Academy of Sciences, Beijing, 100190 China; 2 University of Chinese Academy of Sciences, Beijing, 100049 China; 3 Laser Institute, Qilu University of Technology (Shandong Academy of Sciences), Jinan, 250014 China; 4 Center of Materials and Optoelectronics Engineering, University of Chinese Academy of Sciences, Beijing, 100049 China; 5 The Yangtze River Delta Physics Research Center, Liyang, 213000 China; 6 Songshan Lake Materials Laboratory, Dongguan, 523808 China

**Keywords:** Optoelectronic devices and components, Photonic devices

## Abstract

Mechanically flexible optoelectronic devices and systems can enable a much broader range of applications than what their rigid counterparts can do, especially for novel bio-integrated optoelectronic systems, flexible consumer electronics and wearable sensors. Inorganic semiconductor could be a good candidate for the flexible PD when it can keep its high performance under the bending condition. Here, we demonstrate a III–V material-based flexible photodetector operating wavelength from 640 to 1700 nm with the high detectivity of 5.18 × 10^11^ cm‧Hz^1/2^/W and fast response speed @1550 nm by using a simply top-to-down fabrication process. The optoelectrical performances are stable as the PDs are exposed to bending cycles with a radius of 15 mm up to 1000 times. Furthermore, the mechanical failure mode of the PD is also investigated, which suggests that the cracking and delamination failure mode are dominant in bending up and bending down direction, respectively. Such a flexible III–V material-based PD and design with stable and high performance could be a promising strategy for the application of the flexible broad spectrum detection.

## Introduction

Emerging application of mechanically flexible optoelectronic devices have flourished in the fields of flexible imaging/displays^1–5^, sensors^6–8^, short-reach data communications^9–11^, solar cell^12–14^ and so on. As an indispensable component of flexible optoelectronic systems, flexible photodetectors (PDs) have been extensively studied and achieved breakthroughs in nanrrow band region photoresponse: ultraviolet (UV), visble (Vis), near infrared (NIR) and broadband photoresponse. A great progress has been achieved for visible or near IR organic-based PDs, but there are several key bottlenecks, such as large bandgap, weak absorption, and poor charge generation in the NIR region, resulting in poor performance^15–17^. In recent years, significant progress has been made in multispectral PDs based on inorganic material combined with organic substrates, which makes full use of the high performance of inorganic materials and the mechanical flexibility of organic materials^18–22^. Flexible UV PDs applied ZnO^23,24^, InGaZnO (IGZO)^25^ and Ga_2_O_3_^26^ with wide bandgap illustrate favorable photoresponse performance and flexibility. The inorganic semiconductor nanowires (NWs)^27–29^ and 2D materials^30^ such as graphene^31–34^ and thin transition metal dichalcogenide (TMDCs)^18,35^ have also been applied in the flexible PDs and great progress have been achieved at the UV to NIR range because of their unique electronic structure, easy fabrication, broad spectra absorption and flexibility. Especially, the transition metal dichalcogenide (TMDCs) based on organic substrate, such as MoS_2_ on PET, WSe_2_ or MoS_2_/WSe_2_ on paper, provide advanced flexible visible detectors with high responsivity and quantum efficiency, the preparation scheme has low cost and large device preparation area. Moreover, broadband photodetection has been achieved in TMDCs^18,36^, IGZO^37^ and Si-based material^38^, which enhances the absorption of light by the decoration on surface with dissimilar superstructure or nanostructure. However, the flexible photodetection realized with above material is able to covers UV, Vis and NIR, but has no response to short-wave infrared (SWIR). The response time of the PDs mentioned above exceeds millisenconds (ms)^35^, which is incapable to the application in high-speed detection, such as flexible communication equipment, biological sign detection, etc.

As we know, InGaAs alloy is a key component in the active regions of high-speed electronic devices^39^, infrared photodetector^40^ and lasers^41^. The In_x_Ga_1-x_As photodetector could be optimized for any wavelength with in a spectral range of 0.4 μm-3.6 μm. In_0.53_Ga_0.47_As is lattice-matched to InP. The bandgap of In_0.53_Ga_0.47_As is 0.74 eV, covering 1310 nm and 1550 nm low-loss communication application wavelengths. The electrons mobility of In_0.53_Ga_0.47_As is 12,000 cm^2^/Vs, which is closed to 10 times of that of Si. Combined with the current mature manufacturing technology of III–V semiconductor devices, In_0.53_Ga_0.47_As/InP PD has been widely applied and commercialized in the night vision, inspection, agricultural sorting, and so on. Thus, in this work, we have demonstrated the flexible InGaAs membrane PD with the detectivity of 5.18 × 10^11^ cm‧Hz^1/2^/W by lifting off epitaxial layers from their native substrates using a sacrificial layer and transferring them onto a flexible host carrier. A sidewall passivation is applied to simplify InGaAs PD fabrication process, and large-area flexible PDs with operating wavelength from 640 to 1700 nm are obtained, the response speed to SWIR at 1550 nm is fast enough to reach the MHz response level. Furthermore, the optoelectrical performance of such flexible PDs keeps stable in bending state and the physical mechanism is also studied in details. As a III–V materials based flexible PD with the conventional high performance of the rigid PDs, one can envision its role for the operation from visible light to short wavelength IR in the novel bio-integrated optoelectronic systems, flexible consumer electronics and wearable sensors.

## Methods

**Figure 1:**
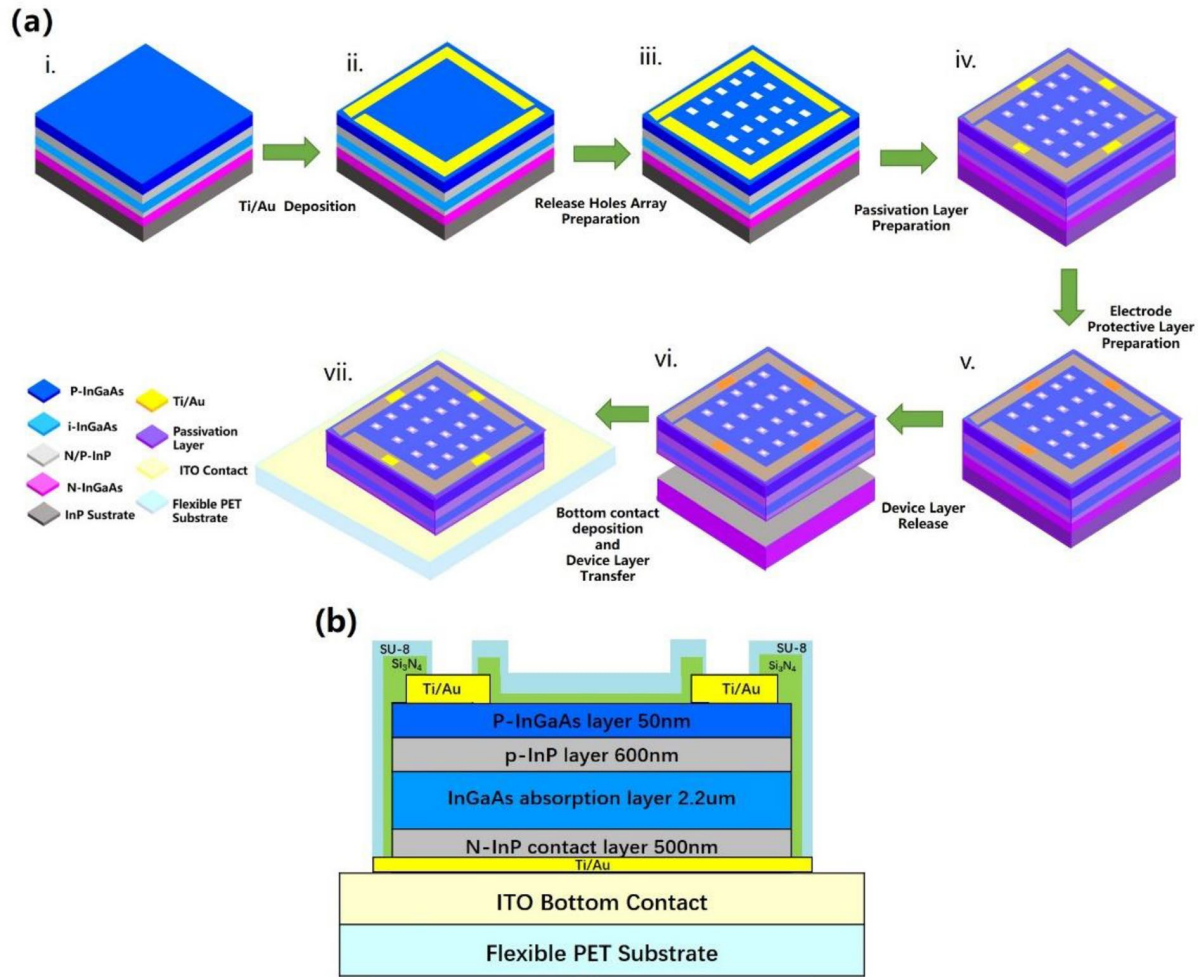
Device fabrication: (**a**) A flow chart for fabrication process: (i) Material growth of InGaAs PIN PD, (ii) Deposition of Up-contact metal frame. (iii) Preparation of release holes array, (iv) Preparation of passivation layer, (v) Preparation of electrode protective layer, (vi) Peeling off the InGaAs PIN membranes, (vii) Deposition of bottom contact and transfer of devices. (**b**) Cross-sectional structure of a fabricated InGaAs PIN flexible PD based on ITO-PET flexible substrate.

The InGaAs PIN flexible PD fabrication process is schematically illustrated in Fig. 1a. The process combines material growth and device fabrication. The process of device fabrication includes seven steps: constructing metal frame, etching out the release holes, fabricating the passivation layer, coating the electrode protective layer, peeling off the membranes, depositing the bottom electrode contact and transferring the device layers to the flexible organic substrate. Details of the flexible PD fabrication and characterization are as follows.

### Material growth

Firstly, the III–V PIN PD membranes were grown on the 2-inch InP substrate by metal organic chemical vapor deposition. The thickness for the p- and n-type InP layers were 600 nm and 500 nm, respectively. There were three lattice-matched In_0.53_Ga_0.47_As layers in the structure, including the top 50-nm-thick p-InGaAs layer as the ohmic contact layer, the 2.2 μm-thick undoped InGaAs layer as the absorption layer, and the 500 nm-thick n-InGaAs layer between n-InP contact layer and n-InP buffer layer as the sacrificial layer. It is noted that the p-type InGaAs layer and InP layer were achieved by single MOCVD diffusion of zinc after the epitaxy. The material characterization of InGaAs PIN photodetector is shown in supplementary material.

### Device fabrication

By using micro/nano fabrication technique, a metal frame layer (20 nm/200 nm Ti/Au) was deposited on the top of the device layers and thus two 150 μm-wide metal bars formed the metal frame layer. The metal frame layer can be used as the supporting frame to increase the mechanical strength of the to-be released InGaAs-based membrane and the top contact layer for the PD devices.

**Figure 2:**
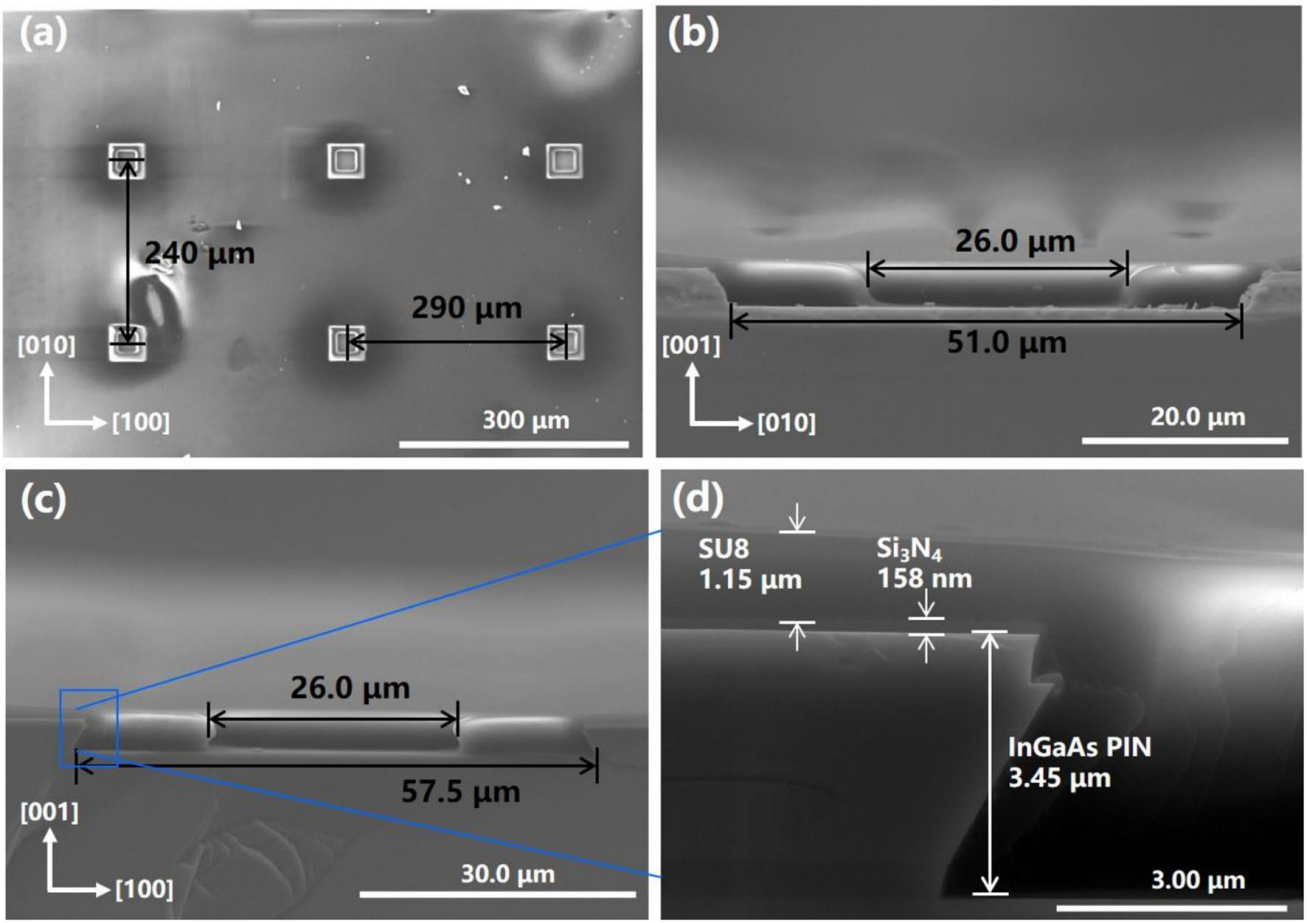
SEM image of etch holes in fabricated InGaAs PD based on the InP substrate: (**a**) The surface of the etch holes array. (**b**,**c**) The cross section of side wall covered with composite passivation layer in [100] and [010] direction. (**d**) A magnified view of a side wall of the outline area on the left shows side wall covered with Si_3_N_4_ (158 nm) and SU-8 (1.15 μm).

Arrays of 40 × 40 μm^2^ square release holes inside were patterned on the device layers in 〈100〉 and 〈010〉 directions, by selectively wet etch down to the surface of the InGaAs sacrificial layer. The release holes were prepared to facilitate the transverse wet etching of InGaAs sacrificial layer beneath the device layer. In the process of selectively wet etching, a H_2_SO_4_:H_2_O_2_:H_2_O (1:8:120) solution selectively corroded InGaAs, but had no effect on InP. On the contrary, the mixed solution of HCl:H_3_PO_4_ (1:3) selectively etched InP but has no effect on InGaAs. Therefore, when InGaAs or InP were etched alternately with different etching solution, excessive etching could be properly performed to ensure sufficient corrosion. Based on the selective wet-etching process, the release holes were formed down to the surface of the InGaAs layer (sacrificial layer). The release holes in detail with scan electron microscopy (SEM) were shown in Fig. 2. As the etching rates of 〈100〉 and 〈010〉 direction were different^42^, the periodic spacing between the release holes was designed to be 240 μm in 〈010〉 and 290 μm in 〈100〉, respectively, as shown in Fig. 2. Compared with the same spacing distances in each directions between the release holes^43,44^, the different distances design ensured that the delamination could be completed simultaneously in both directions in the process of the horizontal wet etching separation. Furthermore, as can be seen in Fig. 2, during the fabrication of release holes, the side length of the square holes expanded from 40 to 57.5 μm in 〈100〉 direction and 51.0 μm in 〈010〉 direction, which causes negligible loss to the photosensitive area.

Since the absorption layer was InGaAs, the same material as the sacrificial layer, the sidewall of the InGaAs active layer in the release holes need to be protected when the selective etching solution flew to the InGaAs sacrificial layer through the release holes and undergoes etching reaction. Thus, a composite passivation layer was applied on the surface and the sidewall of the devices. According to the reference^45,46^, Si_3_N_4_ and SU-8 both play an effective role in improving detector performance, silicon nitride (Si_3_N_4_) can compress dark current to promote the detectivity, SU-8 can provide better mechanical flexible support. Firstly, a 158 nm-thick Si_3_N_4_ layer was deposited on the top of the device layers by ICP-PECVD and then reactive ion etching was performed to open a 40 × 40 μm^2^ square release hole in the center of each release hole formed by wet etching above, intervals between platforms and the top contact under the Si_3_N_4_ layer were also etched out. After that, the devices were coated with 1.15 μm SU-8, and then a 26 × 26 μm^2^ square release hole was opened in the center of each release hole by photolithography, the intervals between platforms and the top contact under the Si_3_N_4_ layer were also exposed again. As shown in the Fig. 2b,c, the shape of release holes coated with SU-8 can be recognized as an inverted trapezoid in 〈010〉 direction and regular trapezoid in 〈100〉 direction. In general, the passivation layer was confirmed to prevent the InGaAs absorption layer from damage during the wet-etching of sacrificial layer.

Ti/Au top contacts were covered with photoresist, a temporary protective layer to prevent the device layers from being damaged by metal-assisted chemical etching effect^47^ during the wet-etching of sacrificial layer, and the photoresist layer would be removed after the transfer. After that, the InGaAs sacrificial layer was selectively etched away by immersing the sample in a H_2_SO_4_:H_2_O_2_:H_2_O (1:8:120) solution, and the metal framed InGaAs PIN detectors detached from the InP host substrate. Subsequently, the flexible membranes were taken out of the etching solution, and slowly dipped into deionized water to remove residual H_2_SO_4_ and H_2_O_2_.

**Figure 3:**
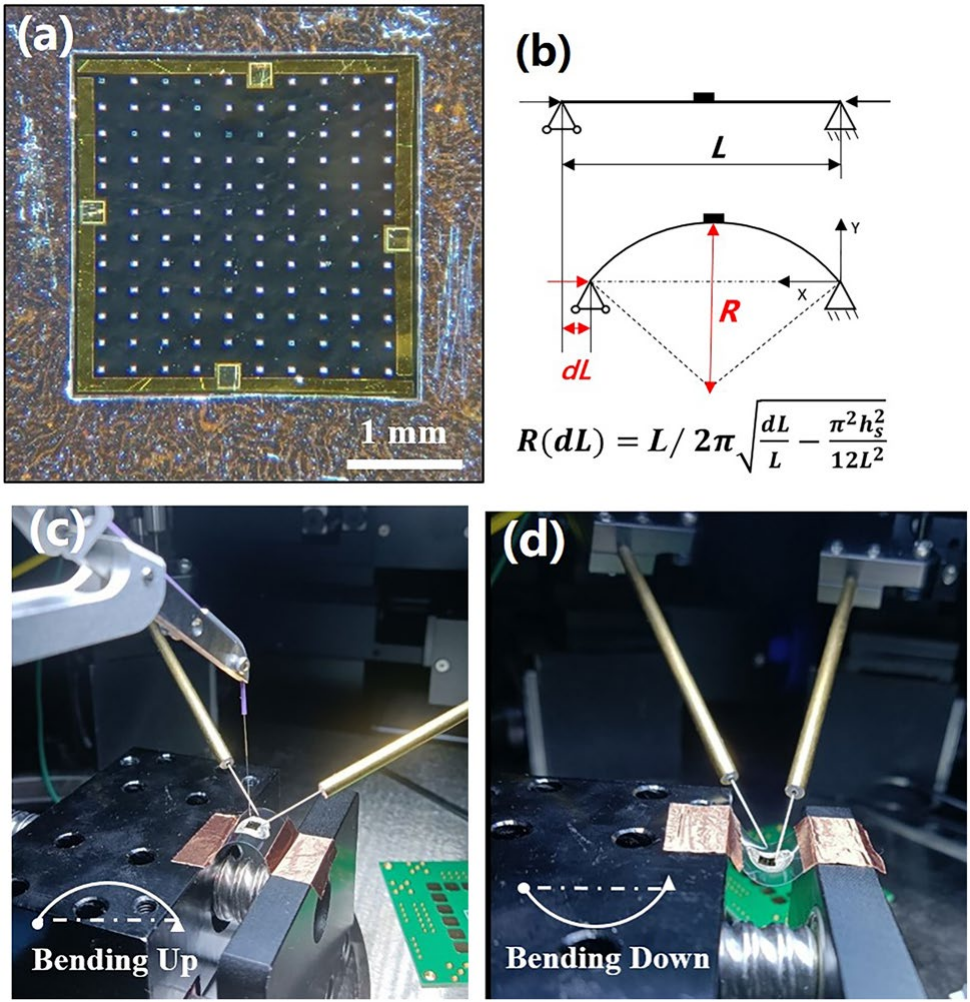
(**a**) Zoom-in views of a fabricated large area (3 × 3 mm^2^) InGaAs PD on flexible PET substrate. (**b**) Schematic diagram for the measurement principle and the corresponding model. (**c**,**d**) A schematic diagram of fabricated flexible InGaAs PD under bending up and bending down test, respectively. The bottom left insets provide schematic illustrations of the bending geometries.

To prepare the bottom contact, the InGaAs PIN flexible detector membranes were flipped and attached to a silicon chip coated with photoresist, then n-contact (20 nm/200 nm Ti/Au) preparation was accomplished by electron beam evaporation. Finally, InGaAs membranes lifted off from the temporary reusable substrate, and a complete InGaAs flexible PD membrane was prepared, which can be installed in any flexible circuit. In our bending experiment, the InGaAs membrane flexible PD were attached to the indium tin oxide (ITO)-PET flexible sheet (0.05 mm thick, length *L* = 35 mm) with silver conductive adhesive (about 1 μm thick), the whole flexible PD structure was shown in Fig. 1b. Micrographs of an actual device was shown in Fig. 3a.

### PD characterization

The InGaAs PIN flexible PD was measured the current–voltage (I–V) in flat state and bent state at different curvature radius(R). The spectral response of the flexible PDs was carried out by a Fourier transform infrared spectrometer (Burker Vertex 70v) at room temperature in a frontside-illuminated format. All current–voltage (I–V) measurements were performed on micromanipulated probe station using a Keithley 4200 semiconductor characterization system at room temperature. An 1550 nm wavelength fiber laser with 2 μm diameter spot was used as a light source for the I-V curves under illumination characteristics. The time resolved optical response of the flexible photodetector is obtained by analyzing the transient response spectrum, the test details and schematic diagram are described in supplementary information. During the bending test, one end of the PET with a flexible PD was fixed and the other end was pushed forward by *dL*, the bending *R* is the function of *dL*^48^, as the model shown in Fig. 3b. The bending tests of devices were performed in two direction: bending up (Fig. 3c) and bending down (Fig. 3d) for tensile and compressive strains, respectively.

## Results and discussion

**Figure 4:**
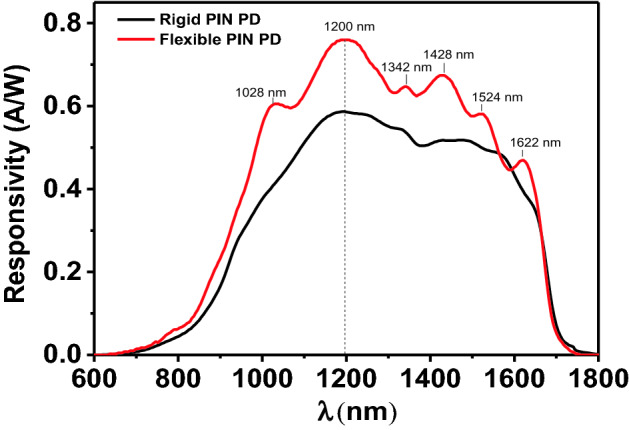
The response spectrum of InGaAs Flexible membrane PD and InGaAs rigid PD at 300 K.

Figure 4 shows the spectral response ranges of the InGaAs-based membrane PD (Red line) and the InGaAs PIN rigid PD (Black line, as a counterpart) with the same area fabricated from the same epitaxy wafer. It can be clearly seen that the response wavelength of InGaAs-based membrane PD is the same with that of rigid PD in the range of 640 nm to 1700 nm, which is consistent with other reports^49,50^. The reason is that the InP contact layer of the flexible InGaAs PIN is 600 nm, and the part of the visible light can pass through the contact layer. According to the previous report^49–51^, if the InP contact layer get thinner, the quantum efficiency in visible light (400–750 nm) and near-IR (750–1000 nm) wavelength region can promote, the PD can detect wavelength as short as 400 nm in the visible region. The peak of the InGaAs PIN flexible PD is 1200 nm, which is consistent with the peak position of InGaAs PIN rigid PD. It is attributed to the stability of InGaAs PIN structure even after peeling off from the InP primary substrate. In addition, peaks at 1028 nm, 1342 nm, 1428 nm, 1524 nm and 1622 nm are considered to be caused by the antireflection effect of the thickness of the SU-8/Si_3_N_4_ composite passivation layer on a specific wavelength, which indicates that the thickness of the passivation layer is uneven and needs to be further optimized. The peak responsivity of the InGaAs PIN flexible PD (0.76 A/W) is 28.8% higher than that of the rigid PD (0.59 A/W), which is due to the reflection of incident light by the Ti/Au bottom contact, thus enhancing the absorption and promotes the application of present device for high resolution photodetection.

**Figure 5:**
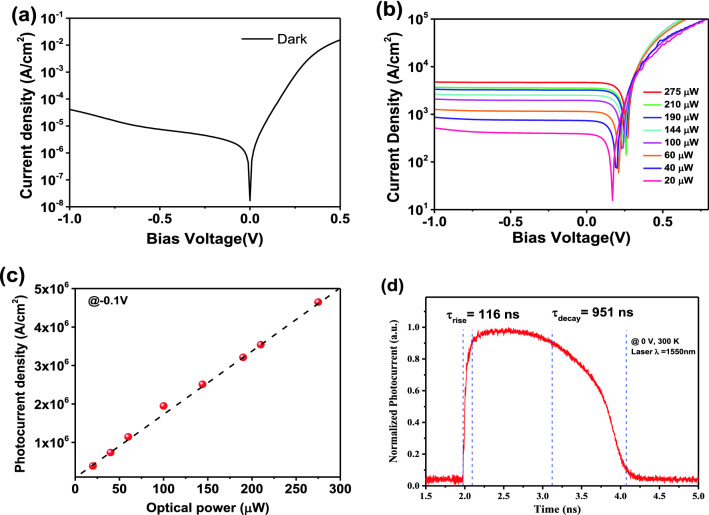
J–V responses of the InGaAs flexible detector under flat condition: (**a**) At darkness and (**b**) Different input optical power levels. (**c**) Photocurrent (PC) as a function of the optical power, the solid line represents a linear fitting of the experimental data. (**d**) Transient response spectrum of the detector under 0 V, inspired by a femtosecond laser (λ = 1550 nm, P = 200 μW with 100 fs pulse width).

Figure 5a,b shows the dark current and photocurrent density (J) of the InGaAs membrane flexible PDs in flat condition under various incident optical powers at 1550 nm. It can be clearly seen that the PDs exhibit typical rectifying characteristics, indicating an excellent quality of the PIN junction. The dark current density is approximately 2.50 μA/cm^2^ at − 0.1 V, and there is a gradual increase of the dark current density at the reverse bias region, which could result from the band-to-band tunneling mechanism^52^. A high sensitivity (i.e., the ratio between dark and photocurrent density) of larger than 1.57 × 10^8^ is achieved at 1550 nm. To obtain the information about inhomogeneities at interface of device, from the slope of the V versus In(J) plot at 300 K in the darkness, the ideality factor n is calculated as followed^36,53^:

1 $$\mathrm{n}=\frac{q}{{k}_{B}T}\frac{dV}{d(\mathrm{ln}(JA))}$$

where *q*, *k*_*B*_, *T*, *J*, and *A* denote electron charge, the Boltzmann constant, operating temperature, dark current density, the effective area of the device, respectively. The ideality factor is 4.46 at 0 V. The value, greater than 1, shows the device suffers from the inhomogeneities, which is due to the poor contact between the bottom Ti/Au contact and ITO.

In addition, the PD exhibits a linear response with the excitation power increasing from 20 to 275 μW, as shown in Fig. 5c, indicating that the photogenerated carriers are dominant^54,55^. The responsivity (R_i_) within the range is 0.53 A/W at − 0.1 V, corresponding to an external quantum efficiency (EQE) of 42.46%. The Johnson noise limited detectivity D* of the device is calculated according to Refs.^56,57^:

2 $${D}^{*}=\frac{q\lambda \cdot EQE}{hc}{\left[2qJ+\frac{4{k}_{B}T}{RA}\right]}^{-\frac{1}{2}}$$

where *q*, *λ*, *h*, *c*, *k*_*B*_, *T*, *J*, *R* and *A* denote electron charge, laser wavelength, the Planck constant, velocity of light, the Boltzmann constant, operating temperature, dark current density, differential resistance and the effective area of the device, respectively, and it can be extracted from the dark current curve that the R_0_A of the membrane PD is 2.37 × 10^5^ Ω cm^2^. As a result, the detector exhibits a room temperature D* of 5.18 × 10^11^ cm‧Hz^1/2^/W @1550 nm at − 0.1 V, whose performance is the same level with the rigid InGaAs SWIR photodetectors^58,59^.

Figure 5d shows the normalized transient response spectrum of the flexible PD, measured at 0 V with 1550 nm laser under a fixed optical power (200 μW). The rise time of the response is 116 ns and the decay time is 951 ns. Due to the slight lattice mismatch or arisen from the InGaAs/InP hetero-interface, two defects with the activation energy of 0.28 eV and 0.15 eV below the conduction band edge are formed and called deep level defect states (DLDS)^60–62^. The DLDS act as additional charge trapping centers of photo generated carriers. As the detectors is illuminated, the electron–hole pairs are generated, then the photo generated electrons are captured in the DLDS before reaching the Ti/Au electrodes. The defect states are charged under illumination until filled, then the electrons in the conduction band are extracted to the electrodes, a new static equilibrium is attained and channel current reaches its maximum value. As the illumination is off, electron trapped in the defect states are released and the equilibrium changes, the channel current decays to its original value^63^. Hence, the process of the defect state filling and the release increase the response time of the flexible detector.

The InGaAs PIN flexible prototype photodetector possesses high surface-to-volume ratio, the surface of the device tend to absorb large number of atmospheric molecular, such as water vapor, oxygen, carbon dioxide absorbed during the device fabrication and testing. The According to the oxygen-assisted mechanism^36,53^:

3 $${O}_{2}\left(g\right)+{e}^{-}\to {O}_{2}^{-}\left(ad\right) (Darkness)$$

4 $${O}_{2}^{-}\left(ad\right)+{h}^{+}\to {O}_{2}\left(g\right) (Illumination)$$

Under the dark condition, the absorbed oxygen atoms in the devices absorb electrons from active material of the device and become negatively charged, as shown in Eq. (3). As the detector is inspired, the photo-generated holes are captured in oxygen absorbed on surface defect states, as shown in Eq. (4), then the holes are released from the oxygen when the illumination is off. As the process of the deep level defect states trapping and releasing photo-generated electrons, the interaction between oxygen and photo-generated holes leads to a prolonged response time. Besides, the high resistance of poor ohmic contact and operating circuit could increase the response time^40^. To shorten the response time of the InGaAs PIN flexible PD, the growth condition and the device fabrication should be optimized further. In general, the results suggest the InGaAs PIN flexible PD is fast enough to reach the MHz response level.

**Figure 6:**
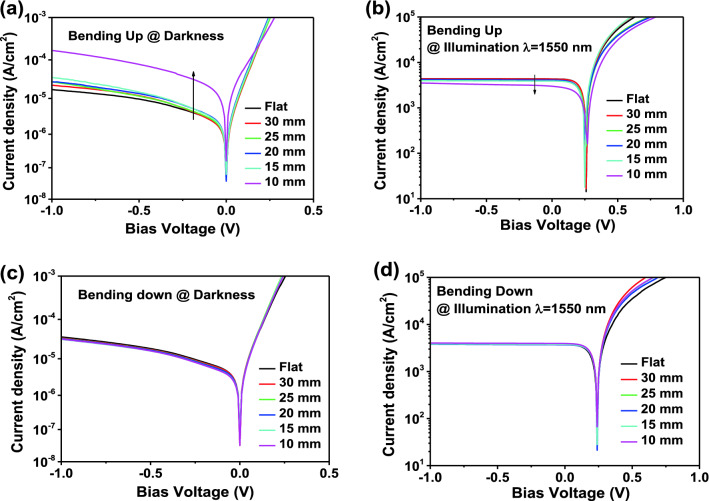
Device characterization under bent conditions: J–V responses of the detector under bending up and down condition. At darkness: (**a**,**c**) and Illumination: (**b**,**d**) at 1550 nm (P = 230 μW).

In order to study the flexible optoelectrical characteristics, the related characterization for the membrane PDs are performed under the bent condition, while the flat condition is referred as the counterpart. Figure 6 shows the PD’s dark current density and photoresponse in their flat and bent up/down state. It should be noted that the dark current density increases gradually under bent down condition (Fig. 6a) as R reduces. Especially when the R is reduced from 15 to 10 mm, the dark current density increases by 5 times. Meanwhile, the phototcurrent density of the device is stable when the radius reduced from 30 to 15 mm, but decreases by 28% as R is 10 mm. Compared with the bending up condition, the dark current and photocurrent both remain stable under bending down condition, as the radius reduces from 30 to 10 mm.

**Figure 7:**
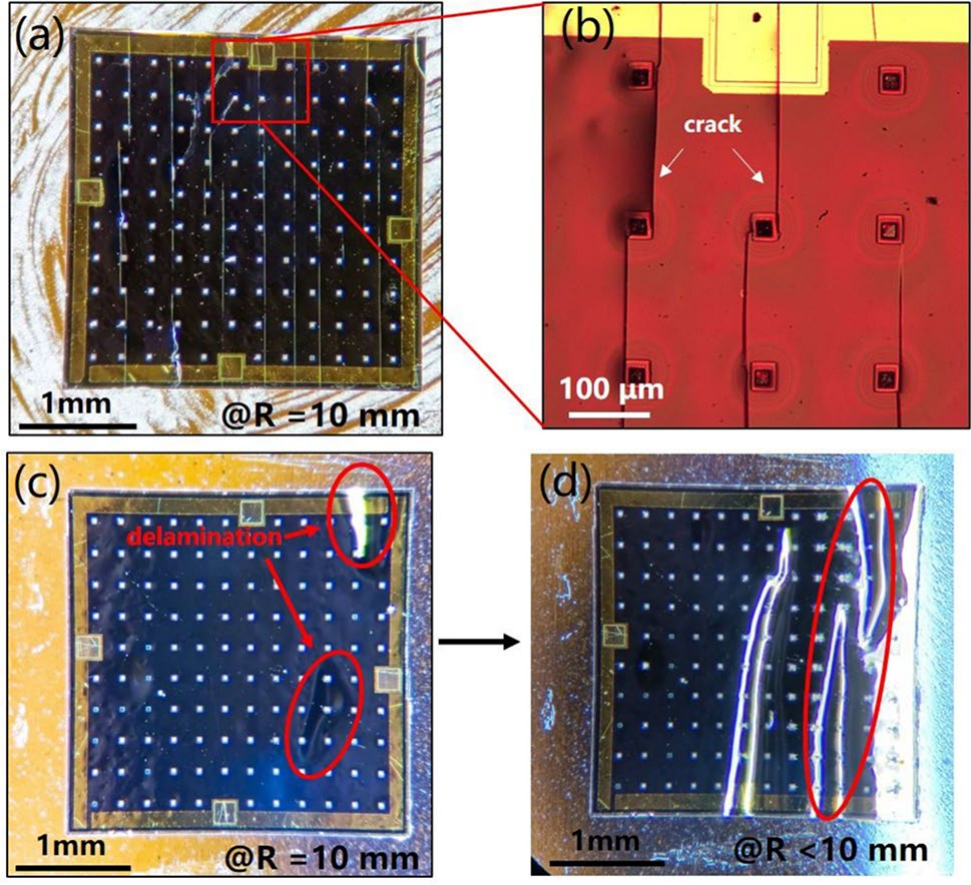
Microscope image of bending flexible InGaAs detector: (**a**) Bending up of R = 10 mm. (**b**) A magnified view of the outline area on the left shows the cracking failure surface. (**c**) Bending down of R = 10 mm shows the appearance of the delamination failure. (**d**) Bending up of R < 10 mm shows the expansion of the delamination failure.

Furthermore, the failure mode of such PDs have been studied deeply. Optical microscope images as shown in Fig. 7 suggest the failure modes of the flexible InGaAs detectors in two bending directions are different. Under bending up condition, cracks are visible at R = 10 mm in Fig. 7a,b, the cracks form perpendicular to the forward direction of *dL* and propagate from one release hole to another one. The cracks lead to the increase of surface leakage current and the decrease of photocurrent. In addition, once the craking mode occurs, the performance degradation is irreversible. In bending down direction, instead of cracking, we observed that the membrane buckled upward via local delamination from the adhesive at R = 10 mm, as shown in Fig. 7c. The delamination reduces the strain in the membrane thereby preventing the cracking failure mode even at smaller bending radius. Therefore, the device can be bent down to R = 10 mm while keeping the integrity and stable photo response of the device. As the bending radius decreases further, the delamination continues to expand, as shown in Fig. 7d. Even if the bending is released, the membranes do not slip back and stay in delamination forever. Instead of the fracture strains of membranes suffering, the interfacial shear stresses are responsible for the failure behavior and the delamination failure is sensitive to the adhesion strength of film to the substrate^48^. Hence, increasing the adhesion strength of InGaAs membrane PD to PET helps to overcome delamination.

According to the study about the failure mode of bending silicon ribbons on plastic substrates, the thickness of the silicon ribbons determines the dominant failure mode of the bending mechanics. As the thickness increases, the dominant failure mode is as follows: cracking, slipping and delamination^48,64,65^. However, there are two failure modes for the InGaAs flexible PD with a fixed thickness in two bending direction. Unlike the silicon ribbon, the InGaAs flexible PD is a composite multilayer asymmetric structure, the effect of bending strain on each layer of material is different. As shown in Fig. 1b, SU-8 layer, Si_3_N_4_ layer and the metal contact coated on the InGaAs PIN membrane structure, asymmetrically from top to bottom. The failure mode is supposed to occur from one layer and transmit to the whole structure. Top and bottom thin metal contact has ductility, and its contribution to the failure membranes can be neglected. The comparison of the Young’s moudlus for SU-8, Si_3_N_4_ and InGaAs PIN membrane is: SU-8 (3–5 GPa) < InGaAs PIN membrane (60–80 GPa) < Si_3_N_4_ (220–230 GPa). The failure mode is more likely to occur in InGaAs PIN membrane and Si_3_N_4_. Considering the thickness of the three materials, it can be assumed that, cracking failure dominated in bending up caused by thin Si_3_N_4_, and the delamination failure dominated in bending down caused by thick InGaAs PIN expitaxy layer. The quantitative analysis of this hypothesis can be further studied, and provide guidance for the bending performance optimization.

In general, the ratio between dark and photocurrent density of the PDs remains stable, larger than 7 × 10^8^ under bending up condition with R decreasing from 30 to 15 mm and bending down condition with R decreasing from 30 to 10 mm, respectively, indicating that the simplify fabrication of InGaAs PIN membrane flexible PD is feasible.

**Figure 8:**
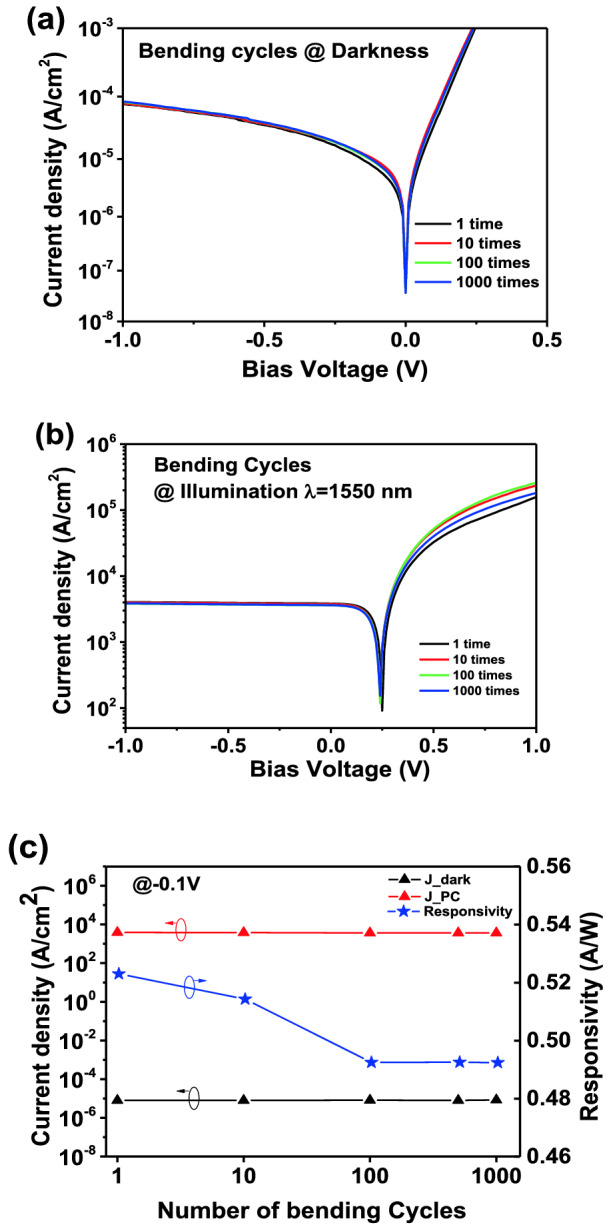
Devices characterization after multiple bending down cycle of 1, 10, 100, 1000 times (a radius of R = 15 mm): (**a**) Dark density of the devices; (**b**) Photocurrent density of the devices; (**c**) Left: Dark and photocurrent (PC) density of the devices at − 0.1 V; Right: Responsivity of the device at 1550 nm (P = 230 μW) at − 0.1 V.

Fatigue test of the InGaAs-based flexible PD is further performed with a self-assembled slide stable system. The PD is bent on the automated slide rail for 1, 10, 100, and 1000-times bending down cycles, respectively. As shown in Fig. 8a,b, the dark and photo current density of the InGaAs PIN flexible PD are stable after 10, 100 and 1000 times bending cycles. The ratio of photo to dark current density remain more than 4 × 10^8^, while the responsivity remains above 0.49 A/W and thus no failure occur after the bending cycle, as shown in Fig. 8c. These results suggest that such PDs have an excellent durability of the optoelectrical performance after multiple mechanical deformations. Therefore, it could be believed that such gratifying robustness of the InGaAs-based flexible PD with the simplified fabrication process is promising to be applied in the flexible optoelectronic field.

## Conclusion

In this paper, we have demonstrated the InGaAs-based membrane flexible PD with the detectivity of 5.18 × 10^11^ cm‧Hz^1/2^/W by a top-to-down fabrication technique using a sacrificial layer and transferring them onto a flexible host carrier. A side wall passivation with SU-8 and Si_3_N_4_ is applied to simplify InGaAs PD fabrication process. And a large-area flexible PD with operating wavelength from 640 to 1700 nm is obtained and the response speed reaches MHz level. Furthermore, the results reveal that the over-all optoelectrical performance of such flexible PDs keeps stable in bending state. In addition, fatigue test suggests that cracking and delamination modes dominates in bending up and down conditions, respectively. We believe that the demonstrated III–V material-based flexible PD with the high performance of the rigid PD is step forward in merging mature compound semiconductor technology and flexible optoelectronic device with simple design and high performance for broad spectrum detection.

## Supplementary Information

Supplementary Information.

## Data Availability

All data generated or analysed during this study are included in this published article (and its Supplementary Information files).
