# Supplementary material for: High performance visible-SWIR flexible photodetector based on large-area InGaAs/InP PIN structure
Source: Sci Rep. 2022 May 10;12:7681. doi: 10.1038/s41598-022-11946-7 (PMC9090829; doi:10.1038/s41598-022-11946-7)
Supplement: Supplementary file 1 — Supplementary Information. [file 41598_2022_11946_MOESM1_ESM.docx]

Supplementary information for High Performance Visible-SWIR Flexible Photodetector Based on Large-area InGaAs/InP PIN Structure

Xuanzhang Li^1,2^, Junyang Zhang^1,2^, Chen Yue^1,2^, Xiansheng Tang^3^, Zhendong Gao^1,2^, Yang Jiang^1,4^, Chunhua Du^1,4,5^, Zhen Deng^1,4,5,^ *, Haiqiang Jia^1,4,6^, Wenxin Wang^1,4,6^ and Hong Chen^1,4,5,6^

^1^ Key Laboratory for Renewable Energy, Beijing Key Laboratory for New Energy Materials and Devices, Beijing National Laboratory for Condensed Matter Physics, Institute of Physics, Chinese Academy of Sciences, Beijing 100190

^2^ University of Chinese Academy of Sciences, Beijing 100049, China

^3^ Laser Institute, Qilu University of Technology (Shandong Academy of Sciences), Jinan 250014, China

^4^ Center of Materials and Optoelectronics Engineering, University of Chinese Academy of Sciences, Beijing 100049, China

^5^ The Yangtze River Delta Physics Research Center, Liyang 213000, China

^6^ Songshan Lake Materials Laboratory, Dongguan 523808, China

*zhen.deng@iphy.ac.cn

1. Material growth of InGaAs PIN PD on InP substrate

The material for InGaAs PIN photodetector was grown on a n-type (10^17^ cm^−3^) InP wafer in a metal organic chemical vapor deposition reactor. First, a n-InP buffer layer (~10^18^ cm^−3^, thickness >200 nm) was grown to stabilize the wafer surface, followed by a 500nm lattice-matched n-type (~10^18^ cm^−3^) In_0.53_Ga_0.47_As, a 500 nm n-type (~10^18^ cm^−3^) InP layer, 2.2 μm i- In_0.53_Ga_0.47_As layer (~10^16^ cm^−3^), a 600 nm n-type (~10^16^ cm^−3^) InP layer and a 50 nm n-type(~10^16^ cm^−3^) In_0.53_Ga_0.47_As layer, respectively. After the epitaxy, the p-type InGaAs layer and InP layer were achieved by single MOCVD diffusion of zinc. The concentrations of p-InGaAs and p-InP layer were about 10^19^ cm^−3^ and 2×10^18^ cm^−3^, respectively. Figure S1 shows the InGaAs PIN photodetector based on InP substrate.


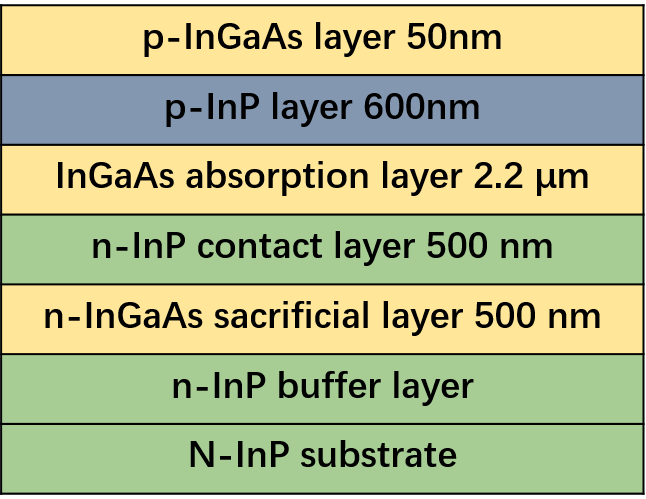


**Figure S1.** The InGaAs PIN photodetector epitaxials structure based on InP substrate

1. Material Characterization

After the epitaxial growth, the material quality was assessed by using high resolution X-ray diffraction (HR-XRD), atomic force microscopy (AFM) and photoluminescence (PL).

Figure S2 shows the HR-XRD of the InGaAs PIN photodetector structure, and Table S1 shows the XRD fitting results of InGaAs PIN photodetector. The HR-XRD ω/2θ curve shows the splitting between InGaAs layer and InP substrate is 108 arcsec and the full width at half maximum (FWHM) value of the InGaAs epilayer is 126 arcsec. According to the Vegard’s law, the In composition of InGaAs can be calculated as 0.536-0.542, the true value depends on the mismatch state between InGaAs and InP substrate. As shown in Figure S3, AFM image shows that the sample exhibited a good surface morphology with clear atomic steps and small roughness of 1.66 Å over a 5 × 5 μm^2^ area. Figure S4 shows the PL curve of InGaAs PIN sample, The peak of curve is 1642 nm (~ 0.755 eV), which is close to the band gap of lattice-matched In_0.53_Ga_0.47_As on InP substrate.

The results from the HR-XRD, AFM, and PL tests illustrate that, the growth conditions of InGaAs PIN structure are appropriate, which is favorable for preparing high-performance detectors.


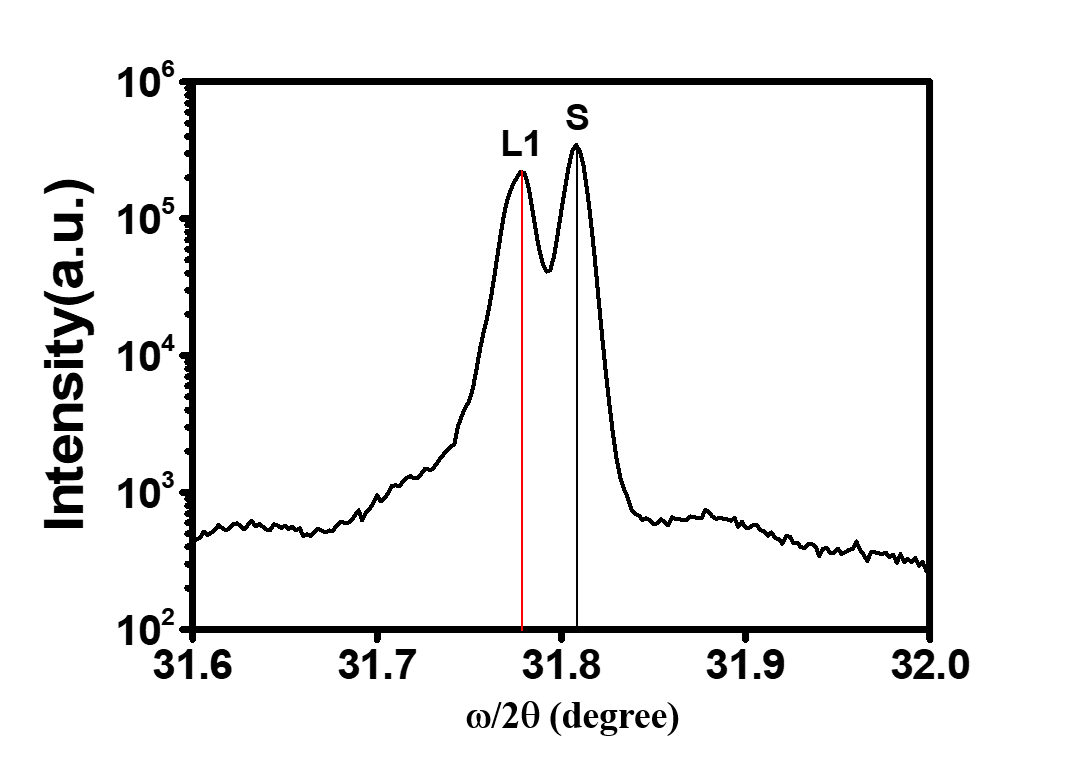


**Figure S2.** the HR-XRD ω/2θ curve of the InGaAs PIN photodetector structure. L1 represents the epitaxy InGaAs layer, and S represents the InP substrate.

**Table S1.** XRD fitting results of InGaAs PIN photodetector

| **Peak Label** | **Name** | **Omega (deg)** | **FWHM（deg）** | **Strained Mismatch（ppm）** | **Strained Composition** | **Relaxed Mismatch (ppm)** | **Relaxed Composition** |
| --- | --- | --- | --- | --- | --- | --- | --- |
| S | **InP** | **31.808** | **0.015** | **——** | **——** | **——** | **——** |
| L1 | InGaAs | 31.778 | 0.014 | 386 | In_0.536_Ga_0.464_As | 770 | In_0.542_Ga_0.458_As |


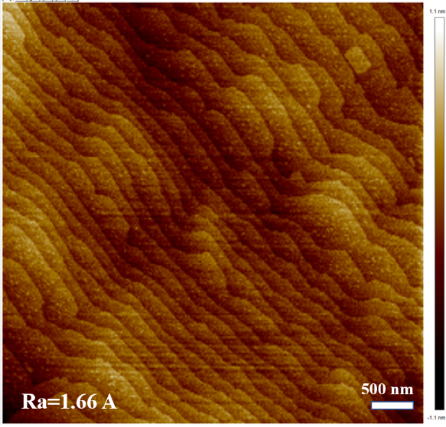


**Figure S3.** The AFM image of a 5 μm × 5 μm surface area of the as-grown material, with rms roughness value of 1.66 Å.


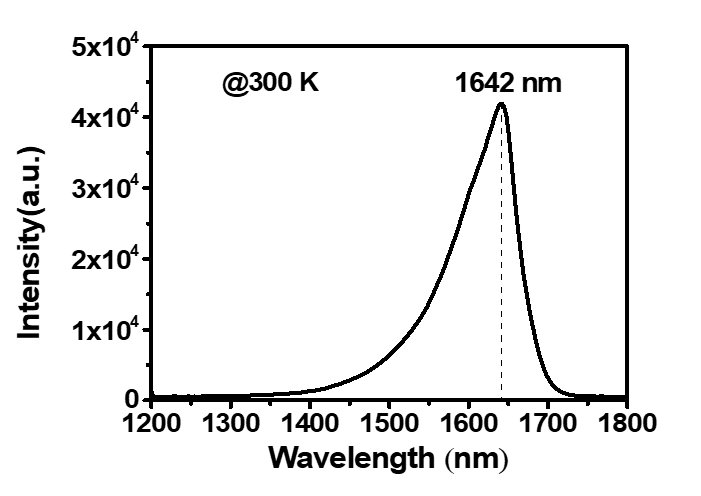


**Figure S4.** PL spectra of InGaAs PIN photodetector structure at room temperature. The peak of curve is 1642 nm (~ 0.755 eV), which is close to the band gap of lattice-matched In_0.53_Ga_0.47_As on InP substrate.

1. The mechanism of the InGaAs PIN flexible photodetector

Essentially, the InGaAs flexible photodetector is a kind of typical PIN infrared photodetector, the energy band structure of the detector is shown in Figure S5. The main InGaAs PIN detector structure consists of 50 nm P-InGaAs, 600 nm P-InP, 2.2 μm InGaAs，500 nm N-InP.

The 2.2 μm InGaAs is the absorption layer of the detector. P-InP and N-InP region form a space-charge field for the absorption layer. The 50-nm P-InGaAs is the ohmic contact layer, which can form low resistance contact with Ti/Au metal so as to lead out photo-generated carriers. Photons with energy greater than the energy gap of InGaAs, incident from the front surface of the device, create electron-hole pairs in the material of the absorption layer. By diffusion, the electrons and holes generated within a diffusion length from the absorption layer reach the space-charge region. Then electron-hole pairs are separated by the strong electric field. The generated carriers are accelerated and extracted from the PIN structure. Through the Ti/Au top contact and the Ti/Au-ITO bottom contact, the photocurrent reaches the external circuit, as shown in Figure S6.


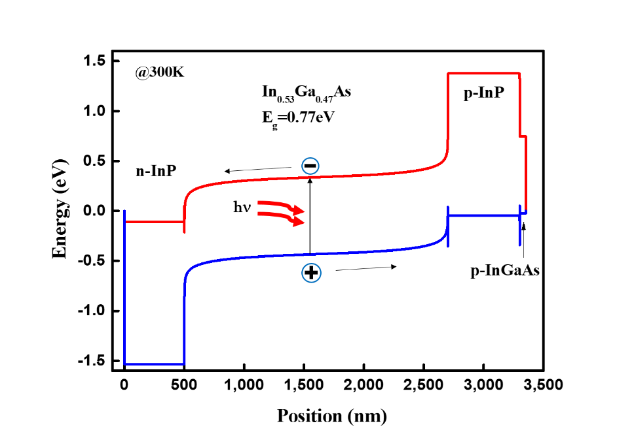


**Figure S5.** Energy band structure of InGaAs PIN flexible photodetector


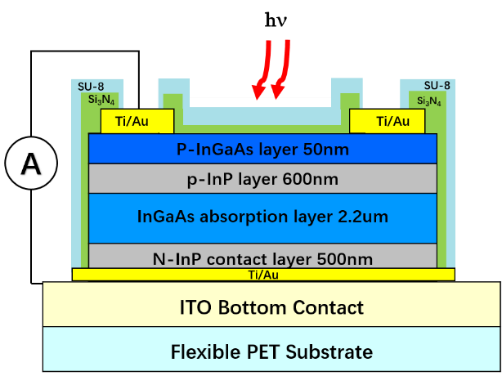


**Figure S6.** Schematic measured diagram of the InGaAs flexible PIN photodetector

1. The transmitted spectrum of passivation layer (Si_3_N_4_ and SU-8)

The Si_3_N_4_, SU-8 and their composite film were prepared on the glass, respectively. Then the samples were put into the UV-Vis-SWIR spectrophotometer, the transmitted spectrum of the sample were attained at the wavelength from 600 to 1800 nm at room temperature. The result is shown in the Figure S7.

The transmittance of Si_3_N_4_ is between 86% and 96 %, the transmission of SU-8 is 98 % to 100 %, the composite film of Si_3_N_4_ and SU-8 is between 88 % and 102 %. The fluctuation of transmittance is due to the interface diffraction between films.

**Figure S7.** Transmitted spectrum of Si_3_N_4_ and SU-8

1. The transient response spectrum system of the InGaAs PIN flexible photodetector

As shown in Figure S8, the InGaAs PIN flexible PD is connected in series with a resistor (R = 50 Ω) in the circuit. The flexible PD is inspired by the femtosecond amplifier laser system (Spectra-Physics), the laser λ=1550nm, power is 200 μW with 100 fs pulse width. As the PD is inspired, the oscilloscope (Tektronix DPO4104 digital phosphor oscilloscope, 1 GHz) records the change of current signal of the resistor with time. In this work, we measured the rise and decay time of the detector as the time taken for the device to reach 90% and 10% of equilibrium value from the initial current, and final current.


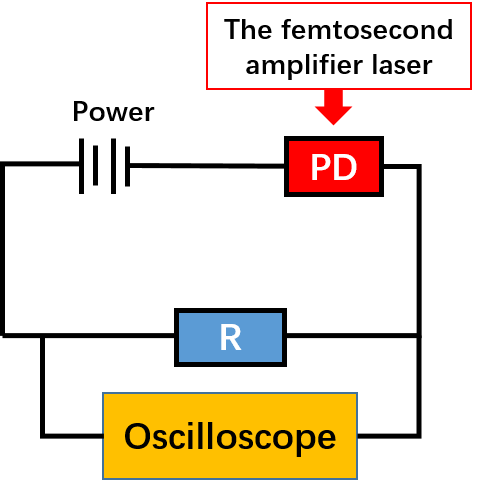


**Figure S8.** Schematic diagram of transient response spectrum system

1. The comparison of the Key Parameters of Flexible Photodetector Found Here and in Earlier Studies

Table S2 summarized the key parameters of flexible photodetector in this study in addition to a comparative analysis with the flexible photodetector report in the earlier literatures.

Table S2: The comparison of the Key Parameters of Flexible Photodetector Found Here and in Earlier Studies

| Materials | Device Architecture | Response spectrum | Responsivity  (A/W) | Respons time  (τ_rise_/τ_decay_) | D*  (Jones) | Dark current density | Max bending radius | Ref |
| --- | --- | --- | --- | --- | --- | --- | --- | --- |
| This study | **InGaAs/InP**  **PIN** | **Visible-SWIR(1.7μm)** | **0.53 A/W**  **@1550 nm** | **116 ns/ 951 ns** | **5.18×10^11^**  **@ 1550 nm** | **7.46 μA/cm^2^ @-0.5 V** | **15 mm** | **—** |
| InP | **InP PIN** | **Visible-NIR** | **10 mA/W**  **@980 nm** | **—** | **5.3×10^9^**  **@980 nm** | **11 μA/cm^2^ @-0.5 V** | **38.1 mm** | **[1]** |
| b-Si/Si | **Al/b-Si/Si**  **N^+^-N** | **Visible-SWIR(1.2μm)** | **63.79A/W**  **@870 nm** | **68 μs / 172 μs** | **1.66 × 10^13^**  **@** | **28 μA/cm^2^ @-0.5 V** | **9.7 mm** | **[2]** |
| Ge | **Ge PIN** | **Visible-SWIR(1.7μm)** | **52.5mA/W @1550 nm** | **2.2 ms / 1.8 ms** | **1.2×10^9^**  **@1550 nm** | **6 mA/cm^2^ @-0.5 V** | **30 mm** | **[3]** |
| WSe_2_ | **WSe_2_ MSM** | **Visible-NIR** | **17.78 mA/W**  **@ 670 nm** | **0.68 s / 1.01 s** | **5.86×10^10^**  **@ 670 nm** | **5 μA/cm^2^ @5V** | **5 mm** | **[4]** |
| PVK/IGZO | **Au/ITO/PVK/IGZO/AU phototransistor** | **UV-NIR** | **3.1×10^5^ A/W**  **@457 nm** | **6 s/—** | **5.1×10^16^**  **@457 nm** | **—** | **Flexibility** | **[5]** |
| MoS_2_ | **MoS_2_ MSM** | **Visible** | **20 mA/W**  **@532 nm** | **12s / 19s** | **—** | **—** | **—** | **[6]** |
| Graphene/MoS_2_ | **Au/PE/SLG/MoS_3_/PET**  **MIS** | **Visible** | **45.5A/W**  **@642 nm** | **—** | **—** | **—** | **14 mm** | **[7]** |
| Ga_2_O_2_ | **Ga_2_O_2_/Ga/Ga_2_O_2_ MSM** | **UV** | **2.2 A/W**  **@254 nm** | **-/<50 ms** | **—** | **2.7x10^-11^A/cm^2^ @10 V** | **8 mm** | **[8]** |

**Reference**

1. Weiquan Yang, Hongjun Yang, Guoxuan Qin, Zhenqiang Ma, Jesper Berggren, Mattias Hammar, Richard Soref, and W. Zhou, "Large-area InP-based crystalline nanomembrane flexible photodetectors," Applied Physics Letters (2010).

2. X. Jin, Y. Sun, Q. Wu, Z. Jia, S. Huang, J. Yao, H. Huang, and J. Xu, "High-Performance Free-Standing Flexible Photodetectors Based on Sulfur-Hyperdoped Ultrathin Silicon," ACS Appl Mater Interfaces **11**, 42385-42391 (2019).

3. S. An, S. Wu, K.-H. Lee, C. S. Tan, Y.-C. Tai, G.-E. Chang, and M. Kim, "High-Sensitivity and Mechanically Compliant Flexible Ge Photodetectors with a Vertical p–i–n Configuration," ACS Applied Electronic Materials **3**, 1780-1786 (2021).

4. P. Pataniya, C. K. Zankat, M. Tannarana, C. K. Sumesh, S. Narayan, G. K. Solanki, K. D. Patel, V. M. Pathak, and P. K. Jha, "Paper-Based Flexible Photodetector Functionalized by WSe2 Nanodots," ACS Applied Nano Materials **2**, 2758-2766 (2019).

5. S. Wei, F. Wang, X. Zou, L. Wang, C. Liu, X. Liu, W. Hu, Z. Fan, J. C. Ho, and L. Liao, "Flexible Quasi-2D Perovskite/IGZO Phototransistors for Ultrasensitive and Broadband Photodetection," Adv Mater **32**, e1907527 (2020).

6. F. Yu, M. Hu, F. Kang, and R. Lv, "Flexible photodetector based on large-area few-layer MoS2," Progress in Natural Science: Materials International **28**, 563-568 (2018).

7. D. De Fazio, I. Goykhman, D. Yoon, M. Bruna, A. Eiden, S. Milana, U. Sassi, M. Barbone, D. Dumcenco, K. Marinov, A. Kis, and A. C. Ferrari, "High Responsivity, Large-Area Graphene/MoS2 Flexible Photodetectors," ACS Nano **10**, 8252-8262 (2016).

8. Y. Sui, H. Liang, W. Huo, Y. Wang, and Z. Mei, "A flexible and transparent β-Ga2O3 solar-blind ultraviolet photodetector on mica," Journal of Physics D: Applied Physics **53**(2020).
